# The estimates of effective population size based on linkage disequilibrium are virtually unaffected by natural selection

**DOI:** 10.1371/journal.pgen.1009764

**Published:** 2022-01-25

**Authors:** Irene Novo, Enrique Santiago, Armando Caballero

**Affiliations:** 1 Centro de Investigación Mariña, Universidade de Vigo, Facultade de Bioloxía, Vigo, Spain; 2 Departamento de Biología Funcional, Facultad de Biología, Universidad de Oviedo, Oviedo, Spain; University of Wisconsin–Madison, UNITED STATES

## Abstract

The effective population size (*N*_*e*_) is a key parameter to quantify the magnitude of genetic drift and inbreeding, with important implications in human evolution. The increasing availability of high-density genetic markers allows the estimation of historical changes in *N*_*e*_ across time using measures of genome diversity or linkage disequilibrium between markers. Directional selection is expected to reduce diversity and *N*_*e*_, and this reduction is modulated by the heterogeneity of the genome in terms of recombination rate. Here we investigate by computer simulations the consequences of selection (both positive and negative) and recombination rate heterogeneity in the estimation of historical *N*_*e*_. We also investigate the relationship between diversity parameters and *N*_*e*_ across the different regions of the genome using human marker data. We show that the estimates of historical *N*_*e*_ obtained from linkage disequilibrium between markers (*N*_*e*LD_) are virtually unaffected by selection. In contrast, those estimates obtained by coalescence mutation-recombination-based methods can be strongly affected by it, which could have important consequences for the estimation of human demography. The simulation results are supported by the analysis of human data. The estimates of *N*_*e*LD_ obtained for particular genomic regions do not correlate, or they do it very weakly, with recombination rate, nucleotide diversity, proportion of polymorphic sites, background selection statistic, minor allele frequency of SNPs, loss of function and missense variants and gene density. This suggests that *N*_*e*LD_ measures mainly reflect demographic changes in population size across generations.

## Author summary

The inference of the demographic history of populations is of great relevance in evolutionary biology. This inference can be made from genomic data using coalescence methods or linkage disequilibrium methods. However, the assessment of these methods is usually made assuming neutrality (absence of selection). Here we show by computer simulations and analyses of human data that the estimates of historical effective population size obtained from linkage disequilibrium between markers are virtually unaffected by natural selection, either positive or negative. In contrast, estimates obtained by coalescence mutation-recombination-based methods can be strongly affected by it, which could have important consequences for recent estimations of human demography.

## Introduction

The effective population size (*N*_*e*_) is a parameter of paramount relevance in evolutionary biology, plant and animal breeding and conservation genetics, because its magnitude reflects the amount of genetic drift and inbreeding occurring in the population [1]. The effective size of a population depends on its demographic history and structure as well as the selection regime affecting the population [2–4]. Estimates of *N*_*e*_ can be obtained by methods using information from genetic markers [3,5,6], and those based on linkage disequilibrium (LD) between them are generally acknowledged to be reliable and robust [7,8]. The idea behind these methods is that, for neutral loci in an isolated population LD is inversely proportional to both the genetic distance (or recombination rate, *c*) between marker sites and the effective size of the population [9].

With the increasing availability of high-density marker information, such as that of single nucleotide polymorphisms (SNP) panels and whole genome sequences for more and more species [10], methods based on LD that allow an estimate of the temporal changes of *N*_*e*_ in the recent past have been developed [11–13]. The basic idea is that LD between pairs of SNPs at different genetic distances provides differential information on *N*_*e*_ at different time points in the past. Thus, Hayes and colleagues [11] suggested that LD between loci with a recombination rate *c* approximately reflects the ancestral effective population size 1/(2*c*) generations ago. Thus, they proposed to estimate *N*_*e*_ at a given generation *t* from pairs of SNPs at a genetic distance 1/(2*t*) Morgans. This method has become increasingly popular for estimating the past and present *N*_*e*_ in human [12,14] and livestock [15,16] populations, and a number of bioinformatic tools have been developed to allow its implementation (e.g. Hollenbeck et al. [17]).

The original application of the above method for estimating historical *N*_*e*_ is, however, restricted to the assumption of constant or linear population growth or decline [11]. Thus, drastic population size changes such as bottlenecks or sudden severe declines in census size, which are common in natural populations or at the start of breeding programs, cannot be detected accurately with this method. A late development has been shown to accurately detect drastic changes in historical *N*_*e*_ (software GONE) [13]. Over relatively recent timespans of about 200 generations back in time, the method has been shown to be more accurate than other alternative coalescence and mutation-recombination-based methods, such as MSMC [18] and Relate [19], which are expected to be applied for long term evolutionary estimations.

All methods of estimation of past *N*_*e*_ trajectories assume neutrality (absence of selection). In this situation, *N*_*e*_ can be estimated from the mean and variance of progeny numbers contributed by parents (*N*_*eVk*_). This value depends on many factors, such as the number of breeding males and females, changes in census size across generations, system of mating, overlapping generations, etc. [1–4], and reflects the amount of neutral genetic drift affecting the whole genome. For a close population with stable demography and breeding system, *N*_*eVk*_ becomes constant over generations. Under selection, however, *N*_*e*_ (which always refers to neutral loci) gets reduced over generations because the cumulative effect of selection on genetic drift, to reach an asymptotic value which is lower than *N*_*eVk*_ [2,20,21]. This reduction occurs even with free recombination and it can be large under artificial selection, as shown initially by Robertson [20]. Under natural selection, the effect is not expected to be so large as for artificial selection except when there is tight linkage [2,22–27]. Thus, under selection and linkage, *N*_*e*_ is lower than *N*_*eVk*_, so that the genetic drift ascribed to neutral genes is higher than that quantified by *N*_*eVk*_.

Natural populations are predicted to encode many deleterious variants [28,29] that can affect the outcome of *N*_*e*_. The fate of these variants, as well as of that of advantageous ones, also relies on linkage, because selection is less effective in genomic regions of low recombination [30]. Genomes are also heterogeneous for genetic variation due to differences in recombination rates across chromosomal regions [31–34] and because of the differential impact of natural selection on them [35]. For example, selective sweeps of favorable mutations are expected to hitch-hike close-by neutral SNPs producing sharp decreases in diversity in linked regions [36–38]. Thus, because the reduction in *N*_*e*_ depends on the recombination rate and the intensity of selection, and these are variable across the genome, the amount of genetic drift for neutral genes is not expected to be the same in different genomic regions, what is called genomic heterogeneity for *N*_*e*_ [2,39–41]. The distribution of genetic variability, both within and between genomes, is affected by the impact of selection on genetic drift and, in particular, nucleotide diversity can be strongly reduced by selection when linkage is tight. Ignoring the heterogeneity in *N*_*e*_ may lead to biased estimates of past demography [42].

It has been shown that selective sweeps of favourable mutations generate LD between close-by neutral loci [43], although this LD increase is transient [38–44] and may be small [45]. In this paper we assess the impact of selection on the estimates of historical *N*_*e*_ obtained by GONE [13], which is based on linkage disequilibrium between SNP markers, in comparison with other coalescence mutation-based methods, MSMC [18] and Relate [19]. Using individual-based forward simulations, we compare the estimates of historical *N*_*e*_ provided by these methods assuming selective sweeps of favourable mutations and background selection on deleterious mutations, and considering the heterogeneity in recombination rates across the genome. We also consider a model of heterozygote advantage for fitness and another with partial self-fertilization, assuming or not selection. In addition, we investigate the relationship between the estimates of linkage disequilibrium *N*_*e*_ and other diversity and genomic parameters across the human genome, using SNP data obtained from genome sequencing of Finnish [46] and Koryaks [47] populations. We obtained the correlation between the estimates of local *N*_*e*_ and several diversity variables over small windows across the genome. Both the simulation results and the analyses of human genome data provide strong evidence that estimates of effective population size based on LD are virtually unaffected by selection.

## Results

### Effect of selection and recombination on the estimation of *N*_*e*_

**Fig 1:**
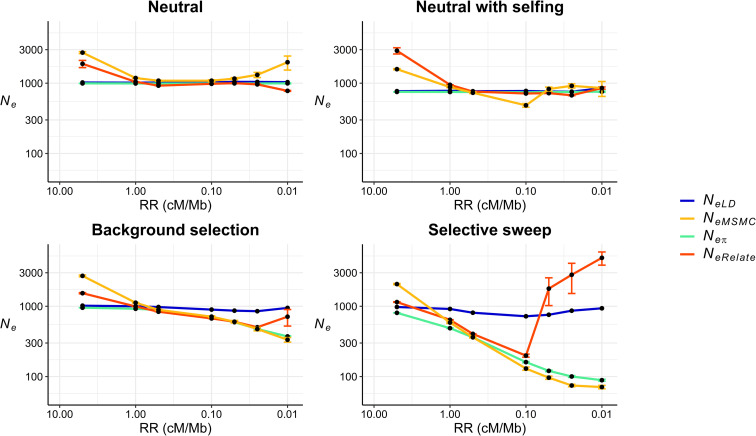
Estimates of effective population size from linkage disequilibrium (*N*_*eLD*_, sample size of *n* = 100 individuals), Relate (*N*_*eRelate*_, *n* = 100), MSMC (*N*_*eMSMC*_, *n* = 4), and from nucleotide diversity (*π*), this latter calculated as *N*_*eπ*_ = *π*/(4*μ*), where *μ* is the nucleotide mutation rate, for scenarios with different recombination rates (*RR* in cM/Mb) uniform across the genome. Simulations assume a fixed population size of *N* = 1,000 individuals under neutrality (random mating or partial self-fertilization with a frequency of 50% selfed progeny), background selection and selective sweeps (both for random mating populations), with constant mutation rate *μ* = 10^−8^ per base per generation. Estimates were obtained including windows of recombination rate between pairs of SNPs ranging from *c* = 0.0025 to 0.0250 for *N*_*eLD*_ and averaging historical estimates of *N*_*e*_ between generations 150 to 350 for *N*_*eRelate*_ and *N*_*eMSMC*_. Error bars represent one standard error above and below the mean of the simulation replicates. *N*_*eLD*_ estimates were obtained pooling all replicates to speed up computation. All simulations were run for up to 100 replicates.

Fig 1 shows the joint effect of recombination and selection on the estimates of *N*_*e*_, for a population of invariable census size of *N* = 1,000 individuals assuming different recombination rates (*RR*) per Mb across the genome. Under a neutral scenario, linkage disequilibrium estimates by GONE (*N*_*eLD*_) provide virtually unbiased estimates of the expected effective population size from the variance of progeny numbers (*N*_*eVk*_) for all recombination rates. In the random mating scenario, *N*_*eVk*_ = *N* = 1,000, the number of breeding individuals. For partial self-fertilization with a proportion 0.5 of selfed mating, *N*_*eVk*_ = 3*N*/4 = 750. The same results can be observed, as expected, for estimates of *N*_*e*_ based on nucleotide diversity (*π*) and calculated as *N*_*eπ*_ = *π*/(4*μ*), where *μ* is the nucleotide mutation rate, assumed also to be constant across the genome. Estimates obtained from Relate (*N*_*eRelate*_) and MSMC (*N*_*eMSMC*_) give also accurate estimates of *N*_*eVk*_ except for the most extreme cases of recombination rate 5 or 0.01 cM per Mb.

Under background selection and selective sweep models in random mating populations, *N*_*eLD*_ estimates give basically the same results as for the neutral model (with some minor deviations for intermediate recombination rates), indicating that these estimates are very little or not affected by selection, either negative or positive (Fig 1). As expected, estimates of *N*_*eπ*_ diverge from *N*_*eVk*_, with decreasing values as recombination rate decreases. Relate and MSMC estimates show a pattern similar to that of *N*_*eπ*_ but *N*_*eRelate*_ estimates increase sharply for tight linkage scenarios. Note that the lowest recombination rate assumed (*RR* = 0.01) implies a genetic map size of only 1 cM for a genome of 100Mb, so we are evaluating very extreme scenarios of linkage. For the partial self-fertilization model, the results under background selection and selective sweeps are similar to those with random mating (S1 Fig), although *N*_*eLD*_ appears to increase with the hitch-hiking model for extreme cases of tight linkage.

Finally, a model of overdominance for fitness under random mating (S2 Fig) shows that *N*_*eLD*_ is almost unaffected by selection for recombination rates down to 0.05 cM per Mb (a genome size of 5 cM). Below this threshold, there is a sharp reduction in *N*_*eLD*_ caused by the appearance of linkage blocks of mutations in heterozygous state. Nucleotide diversity increases for low recombination rates, as expected with this model, and the same behaviour is observed for the estimates from *N*_*eRelate*_ and *N*_*eMSMC*_.

### Simulation results for historical *N*_*e*_ estimates

**Fig 2:**
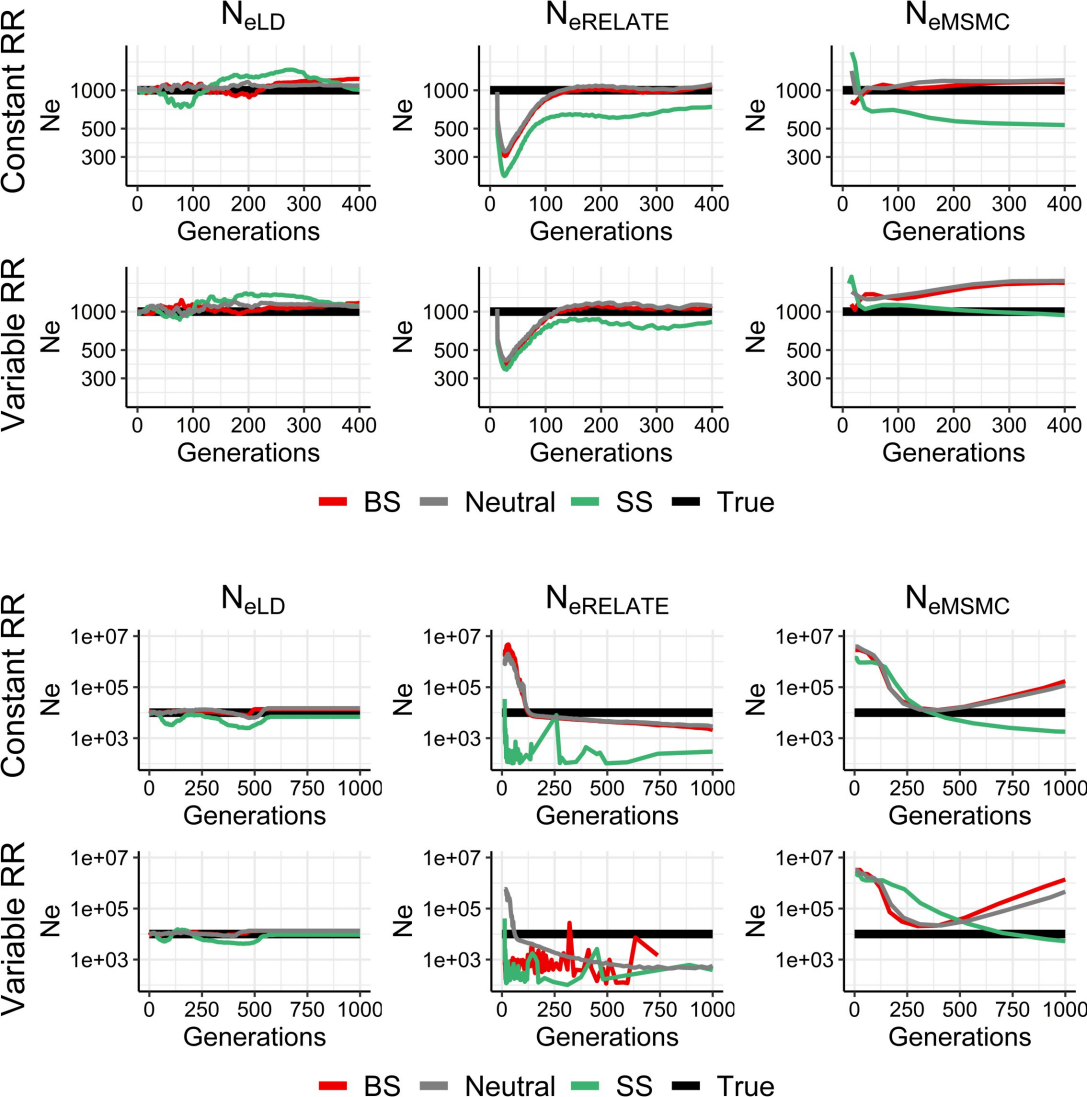
Estimates of historical effective population size from linkage disequilibrium (*N*_*eLD*_, sample size *n* = 100 individuals), Relate (*N*_*eRelate*_, *n* = 100) and MSMC (*N*_*eMSMC*_, *n* = 4) from the present generation (generation 0) back to 400 or 1,000 generations in the past. 100 replicates were run of simulations with constant population size (*N*) under random mating and neutrality (grey), background selection (BS, red) or selective sweeps (SS, green), with constant (1 cM/Mb) or variable recombination rates (*RR*), and constant mutation rate *μ* = 10^−8^ or 10^−9^ mutations per base per generation for *N* = 1,000 or *N* = 10,000, respectively. The true simulated effective size from variance of family size (*N*_*eVk*_ = *N*) is shown in black.

Fig 2 shows estimates of historical *N*_*e*_ assuming an invariable population census size (*N* = 1,000 or 10,000), either with a fixed or a variable recombination rate. Estimates of historical *N*_*eLD*_ reflect the effective population size in the absence of selection (*N*_*eVk*_ = *N*) regardless of selection and the variability in genomic recombination rates. Estimates from *N*_*eRelate*_ and *N*_*eMSMC*_ can give unbiased values of *N*_*eVk*_ under a neutral and background selection model if the initial generations are discarded and population size is not too large (*N* = 1000). Otherwise, they can show important differences from *N*_*eVk*_, particularly under a selective sweep model, where *N*_*eVk*_ is underestimated by *N*_*eRelate*_, and can be either underestimated or overestimated by *N*_*eMSMC*_. In general, variation in the recombination rate across the genome has a limited impact on the estimates of *N*_*eVk*_ with respect to a fixed recombination rate model.

**Fig 3:**
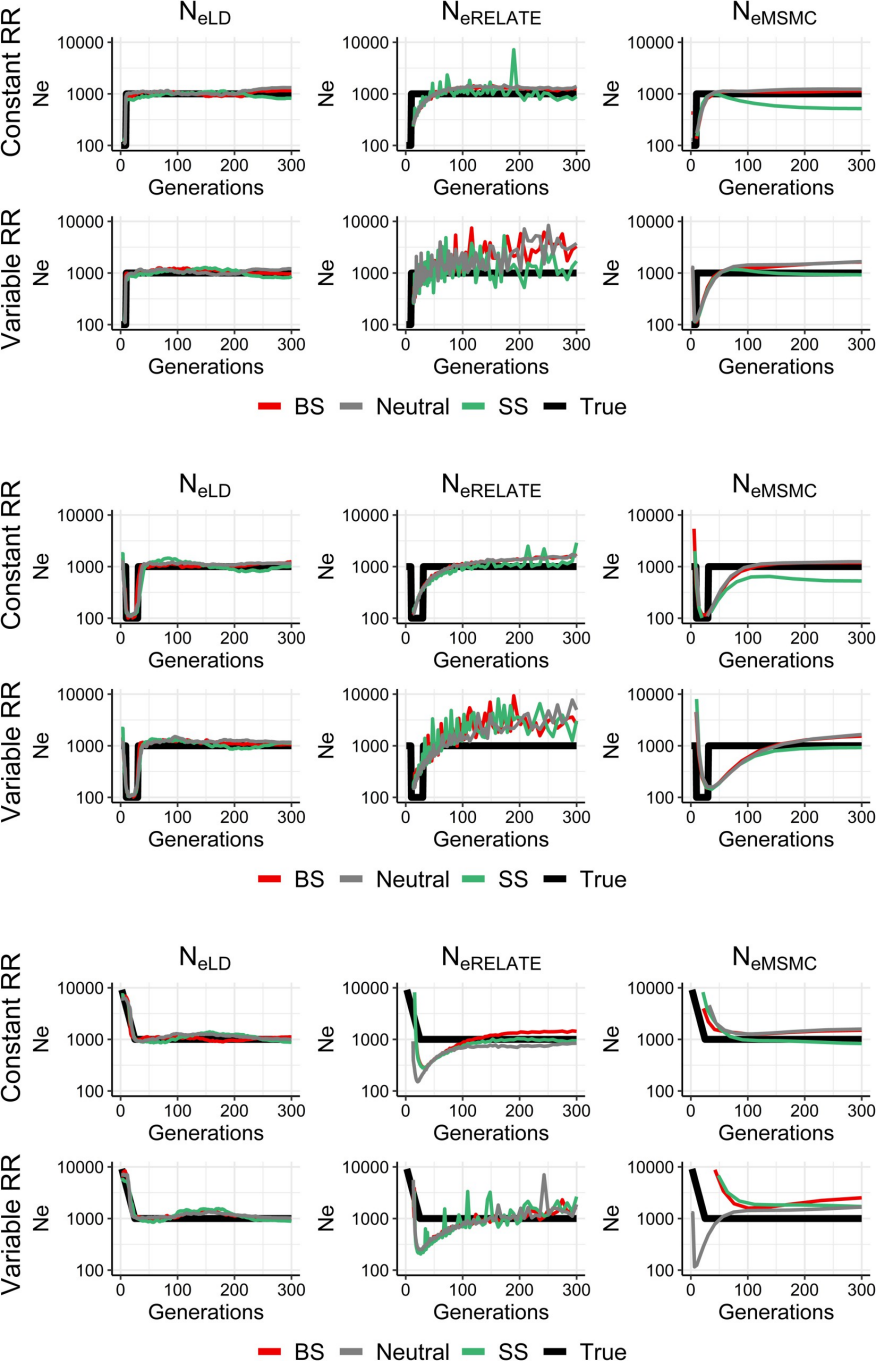
Estimates of historical effective population size from linkage disequilibrium (*N*_*eLD*_, sample size *n* = 100 individuals), Relate (*N*_*eRelate*_, *n* = 100) and MSMC (*N*_*eMSMC*_, *n* = 4) from the present generation (generation 0) back to 300 generations in the past. 100 replicates were run of simulations with constant population size (*N*) under random mating and neutrality (grey), background selection (BS, red) or selective sweeps (SS, green), with constant (1 cM/Mb) or variable recombination rate (*RR*), and constant mutation rate *μ* = 10^−8^ or 10^−9^ mutations per base per generation for *N* = 1,000 or *N* = 10,000, respectively. The true simulated effective size from variance of family size (*N*_*eVk*_ = *N*) is shown in black.

Regarding historical estimates with variable *N*_*e*_ (Fig 3), estimates of *N*_*eLD*_ predict accurately a recent demographic change in population size (occurred 30 generations in the past) regardless of selection and recombination rate heterogeneity. Relate’s estimates show a certain noise, particularly for selective sweeps and/or variable recombination rate scenarios, but give the approximate *N*_*eVk*_ value except for an overestimation in some cases. *N*_*eMSMC*_ estimates are also generally accurate, although they may show some over or underestimations.

**Fig 4:**
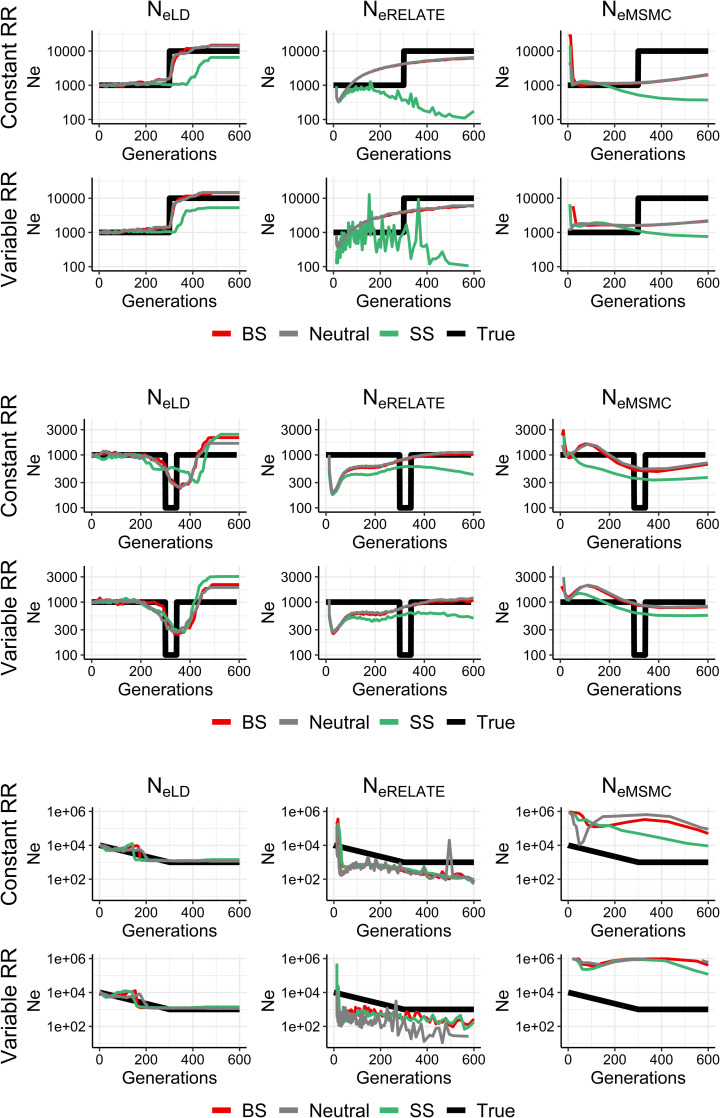
Estimates of historical effective population size from linkage disequilibrium (*N*_*eLD*_, sample size *n* = 100 individuals), Relate (*N*_*eRelate*_, *n* = 100) and MSMC (*N*_*eMSMC*_, *n* = 4) from the present generation (generation 0) back to 600 generations in the past. 100 replicates were run of simulations with constant population size (*N*) under random mating and neutrality (grey), background selection (BS, red) or selective sweeps (SS, green), with constant (1 cM/Mb) or variable recombination rate (*RR*), and constant mutation rate *μ* = 10^−8^ or 10^−9^ mutations per base per generation for *N* = 1,000 or *N* = 10,000, respectively. The true simulated effective size from variance of family size (*N*_*eVk*_) is shown in black.

When population size changes occur in more ancient times (around 300 generations ago; Fig 4), both *N*_*eRelate*_ and *N*_*eMSMC*_ are unable to detect these demographic changes and appear to be affected by selective sweeps, while *N*_*eLD*_ is generally more accurate and it is not affected by selection. The reason why *N*_*eLD*_ performs better to detect recent rather than ancient changes (cf. Figs 3 and 4) is that the linkage signals induced by drift are lost by recombination at a rate *c* per generation, so that changes occurred in the ancient times are less likely to persist. A variable recombination rate does not substantially affect the estimates.

### Correlation between regional estimates of *π* and *N*_*eLD*_ with other diversity parameters using human data

**Fig 5:**
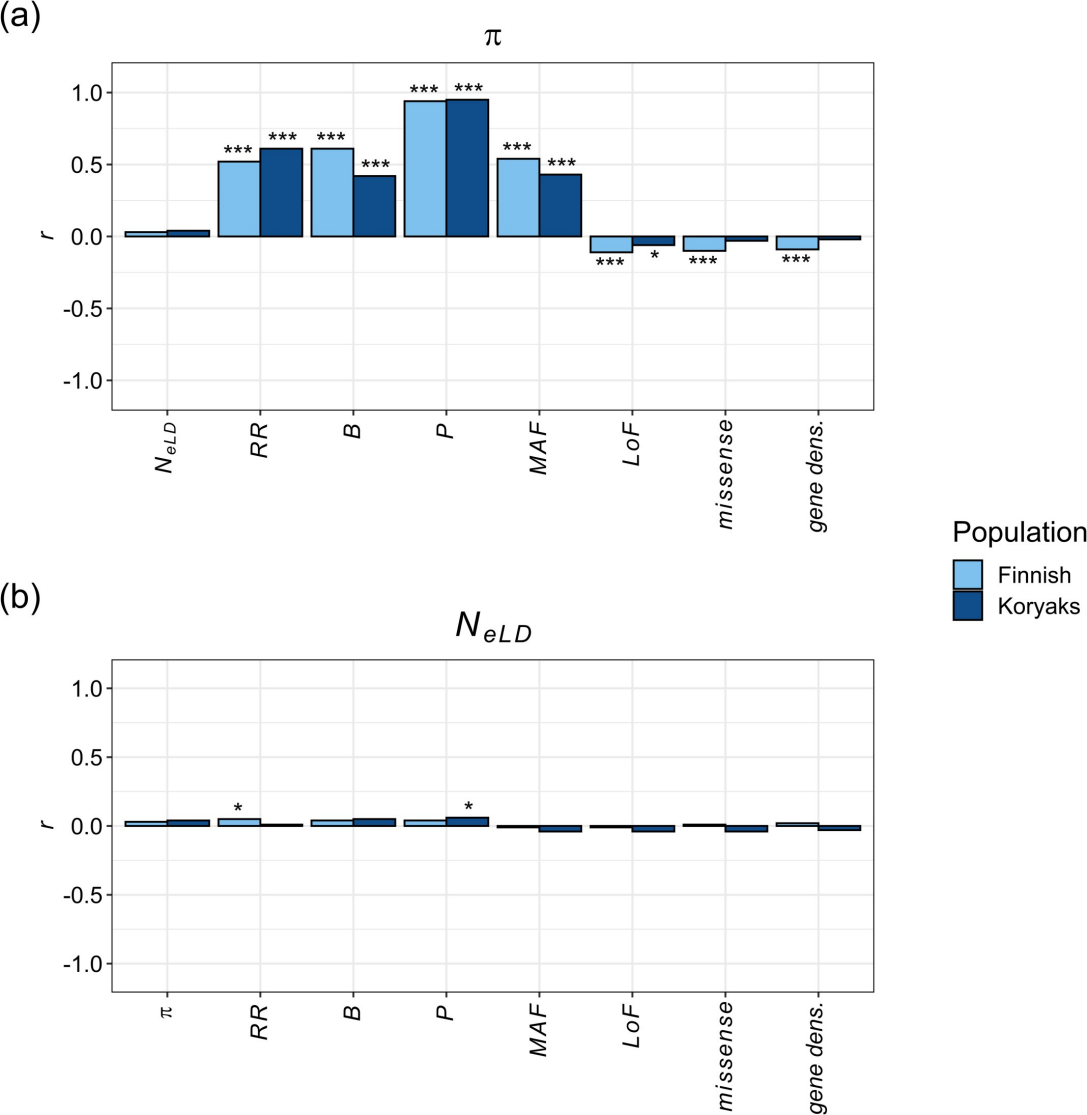
Spearman’s correlation coefficient (*r*) between nucleotide diversity (*π*; panel a) or estimates of linkage disequilibrium effective population size (*N*_*eLD*_; panel b) and different diversity variables, estimated within genomic regions. *RR*: recombination rate; *B*: B statistic; *P*: proportion of polymorphic nucleotides; MAF: Minor Allele Frequency; *LoF*: number of Loss of Function variants; *missense*: number of missense variants; *gene dens*: gene density. Estimates are based on samples of *n* = 99 for Finnish and *n* = 16 for Koryaks populations. P-values: * < 0.05, *** <0.001.

The correlation between the average nucleotide diversity (*π*) in each genomic region and the other related variables followed the expected trends (Fig 5A). A strong positive correlation was found between *π* and recombination rate *RR*, the background selection statistic *B*, the proportion of polymorphic sites *P*, and MAF of SNPs. Nucleotide diversity was also weakly negatively correlated with loss-of-function, missense mutations and gene density, but only significantly for the Finnish population. Finally, no correlation was found between *π* and *N*_*eLD*_.

The correlations among all genetic variables studied are shown in the Supplemental material (S3 Fig), and follow the expected trends. For example, recombination rate *RR*, the *B* statistic, the proportion of polymorphic sites *P*, and MAF of SNPs were highly positively correlated among them. The *B* statistic was strongly negatively correlated with gene density and deleterious variation (LoF, missense), and these latter were highly correlated among them.

The mean, median and standard deviation of the estimates of *N*_*eLD*_ across regions were 11,600, 7,202, and 13,638 for the Finnish population, respectively, and 5,866, 985, and 16,063 for the Koryaks population, respectively. Thus, the standard deviation of the regional estimates of *N*_*eLD*_ relative to a mean of one, for comparison, were 1.18 for the Finnish population and 2.74 for the Koryaks population. The distribution of *N*_*eLD*_ values across genomic regions for both populations is shown in S4 Fig.

The correlation between the estimates of *N*_*eLD*_ and other diversity parameters for different genomic regions are shown in Fig 5B. The correlations did not follow the trends observed for nucleotide diversity. There was no significant correlation between *N*_*eLD*_ and the rest of variables except for a small significant correlation between *N*_*eLD*_ and *RR* for the Finnish population and another between *N*_*eLD*_ and *P* for the Koryaks population.

## Discussion

Our results show that the estimates of the effective population size obtained from linkage disequilibrium between pairs of SNPs (*N*_*eLD*_) are virtually unaffected by either positive or negative selection, thus providing estimates of the effective size from the variance of family sizes (*N*_*eVk*_). This has been deduced from simulation data assuming fixed or variable population size and different selection models (Figs 1–4). The simulation results are supported by those obtained from real human genomic data. The estimates of *N*_*eLD*_ in genomic windows are generally uncorrelated or weakly correlated with recombination rate, the *B* statistic (which quantifies the strength of background selection), nucleotide diversity and polymorphism, as well as the number of deleterious variants (loss-of-function and missense variants) and density of genes (Fig 5). Thus, the results show that the estimates of *N*_*eLD*_ are basically unaffected by selection.

Our interest here was to quantify the impact of selection on the estimates of historical *N*_*e*._ Thus, we assumed a relatively large sample size for the analyses (100 individuals) in order to obtain reasonably accurate estimates. Estimates with lower sample sizes generally would produce noisier estimates (as seen before for GONE estimations [13]) and less accurate inferences about the evolutionary history of the population as a whole [48,49]. The MSMC method could not be applied with more than eight haplotypes for practical reasons, so in that sense it has some disadvantage with respect to the other methods. However, the method worked well in many situations even with this low sample size.

We considered the most characteristic models of natural selection (background selection on deleterious mutations and selective sweeps for advantageous mutations). The observed lack of an impact of selection on the estimates of *N*_*eLD*_ occurs for both models, and this was shown both for random mating and partially self-fertilising populations. We also assumed a model of overdominance for fitness. For this model, the nucleotide diversity is increased with tight linkage (S2 Fig), as expected, which contrasts with the empirical evidence showing that nucleotide diversity is generally reduced in regions of low recombination [35]. This reduction can be clearly seen from the human data analysed here (see S5 Fig). The heterozygote advantage for fitness assumed does not affect either the estimates of *N*_*eLD*_ unless the genetic length of the genome is so small (less than 5 cM) that linkage blocks of balanced mutations in heterozygote state are created. This artefact drastically increases linkage disequilibrium and reduces *N*_*eLD*_ (S2 Fig).

Although positive selection is known to generate linkage disequilibrium between neutral loci close to selective loci [43], this effect is transient and may disappear quickly [38,44]. Stephan and colleagues [44] showed that the increase in linkage disequilibrium between two neutral loci occurs if the recombination rate between the selected locus and the neutral loci is less than *c* = 0.1*s*, where *s* is the selection coefficient of the advantageous allele in homozygosis. We performed simulations with an average *s* = 0.02, which implies that LD is generated between loci located at a genetic distance of *c* = 0.002, or 0.2 cM. In the most extreme linkage scenario simulated in Fig 1 we assumed a rate of recombination of *RR* = 0.01 cM per Mb, which implies a total genetic distance of 1 cM for the whole simulated genome sequence of 100 Mb. Thus, in this extreme scenario it is likely that the linkage disequilibrium between many pairs of close-by SNPs can be transitorily affected by selection. However, we found that the estimates of *N*_*eLD*_ appear to be basically unaffected by positive selection even with tight linkage. To explain this result, we should take into account that the estimation of *N*_*eLD*_ is not based only on the linkage disequilibrium between consecutive or close-by SNPs. It is rather based on the linkage disequilibrium between all pairs of loci across the genome. Recent estimates of *N*_*eLD*_ rely more strongly on pairs of SNPs at large genetic distances, whereas old estimates rely more strongly on SNPs at close genetic distances, and the latter are more affected by selection. However, even so, most pairs of SNPs used in the estimation of *N*_*eLD*_ are likely to be far away from selective loci even in the background selection model, where we assumed that only 5% of mutations are deleterious (0.1% in the selective sweep model of advantageous mutations). Therefore, even though a selective locus may have some impact on the linkage disequilibrium of close-by neutral SNPs, the average linkage disequilibrium of all pairs of SNPs is expected to be weakly affected.

In contrast with the above result of a near independence of *N*_*eLD*_ from selection, the effective population size obtained from nucleotide diversity (*N*_*eπ*_) is expected to be drastically reduced in regions of low recombination under selection [2,27,50] (Fig 1). The observed strong correlations between the regional genomic values of *π* and the recombination rate, the background selection statistic, and the deleterious variants from human data (Fig 5A), also confirm this observation. Estimates of *N*_*e*_ obtained by mutation-recombination-based coalescence methods (MSMC and Relate) are also affected by selection (Figs 1–4). In fact, a Relate Selection Test based on estimating the speed of spread of a particular lineage relative to other competing lineages has been proposed [19]. MSMC estimates generally follow the pattern of *N*_*eπ*_ values except for large recombination rates, for which it provides overestimates of the effective population size in the absence of selection (*N*_*eVk*_; Fig 1). Relate estimates also follow *N*_*eπ*_ values down to recombination rates of 0.1 cM/Mb but, for lower rates, the estimates increase drastically above the true population size (Fig 1). These coalescence methods have been used to investigate ancient demography of human populations and are not generally applicable to short-term historical changes in population size (see Figs 2–4). In fact, it has been acknowledged that MSMC with 8 haplotypes works for estimations before about 70 generations in the past, i.e. about 2,000 years for human populations [18], and Relate seems to discriminate before about 1,000 years back [19]. Thus, MSMC was able to detect the out-of-Africa bottleneck in non-African populations from 200,000 years ago until 50,000 years ago [18], while Relate detected it from 40,000 to 20,000 years ago [19]. The possible impact of selection on these demographic inferences is an issue to be further investigated.

The effect of recombination rate heterogeneity on historical *N*_*e*_ estimates is not very noticeable in most cases (Figs 2–4), particularly when GONE and MSMC are used. This is in accordance with the analyses made by Schiffels and Durbin (their S4 Fig) [18], showing that simulated estimates from MSMC obtained using chunks of the human recombination map do not differ much from those using a constant recombination rate. For Relate estimates, recombination rate heterogeneity seems to affect the estimates of recent demographic changes (Fig 3), generating noisier estimations.

Gossmann and colleagues [39] quantified the heterogeneity of *N*_*e*_ across the genome of ten eukaryotic species (including humans) through the nucleotide diversity of genome sites and accounting for the differences in mutation rate between loci by considering the divergence between species. Thus, they obtained estimates of *N*_*eπ*_, finding a modest but statistically significant variability of this parameter for most species. Gossmann and colleagues [39] found that *N*_*eπ*_ was only positively correlated with recombination rate for *Drosophila* (*r* = 0.45), and negatively correlated with gene density for *Arabidopsis* (*r* = –0.11) and humans (*r* = –0.19). These correlations generally agree with those found for nucleotide diversity in our analysis (Fig 5A), i.e., *r* = 0.51 and 0.60 between *π* and *RR*, and *r* = –0.10 and 0.001 between *π* and gene density, for Finnish and Koryaks, respectively. The failure of Gossmann and colleagues [39] to detect further correlations was attributed to the small variation of *N*_*eπ*_ observed or to only having considered neutral diversity (synonymous changes). In another analysis of genome *N*_*e*_ heterogeneity, Jiménez-Mena and colleagues [40] also found significant variation in *N*_*e*_ across the genome of cattle populations using the temporal *N*_*e*_ estimation method, but this variation did not correlate with the recombination rate, the density of genes, or the presence of loci under artificial selection. This negative result was attributed to the assumption of large genomic windows in order to have a large enough number of markers in each of them, or to the fact that the temporal method of estimation of *N*_*e*_ was only based on a single-generation interval [40,41]. It can also be argued that the estimate of *N*_*e*_ obtained by the temporal method is closer to *N*_*eVk*_ than to *N*_*eπ*_, what may also contribute to explain the lack of significant correlations.

The correlations found between the different parameters analyzed with genomic data followed the expected trends (S3 Fig) and agree with previous estimations. For example, analyzing 100 kb windows of a Danish population, Lohmueller and colleagues [51] found significant correlations between the recombination rate and the number of SNPs in the windows (*r* = 0.20), the SNP diversity (*r* = 0.11) and SNP MAF (*r* = 0.062). These correlations are compatible with those found in our study between *RR* and genome diversity parameters (S3 Fig).

In summary, our results show that the estimates of historical effective size obtained from linkage disequilibrium between pairs of SNPs are not substantially altered by selection, either positive or negative, nor are they affected by the heterogeneity in recombination rate across the genome. Therefore, linkage disequilibrium *N*_*e*_ reflects the true demographic changes in population size over generations. In contrast, other methods based on mutation and recombination, from which recent estimates of human demography have been obtained, can be sometimes affected by selection.

## Methods

### Computer simulations

Genomic data of populations under different demographic and evolutionary scenarios were simulated with the software SLiM 3 [52]. This software simulates a Wright-Fisher model of reproduction with the possibility of adding different types of selection and non-random mating. Random mating populations of constant or changing size (ranging between *N* = 100 and 10,000) with discrete generations were run for up to 10,000 generations. Different demographic scenarios (constant population size, bottlenecking, exponential growth, etc.) were assumed. A model of partial self-fertilization (50% of selfed mating) was also simulated. Genome sequences with a length of 100 Mb were considered where mutations occur at a rate between 10^−7^ and 10^−9^ mutations per nucleotide and generation, depending on the demographic scenario and simulation, with different recombination rates (ranging from 5 to 0.01 cM per Mb). For each locus, values of fitness of 1, 1 + *sh*, and 1 + *s* were considered for the wild-type homozygote, the heterozygote, and the mutant homozygote, respectively. Fitness of an individual was assumed to be multiplicative across loci. Four mutation models were assumed. (1) A neutral model for all mutations (*s* = 0). (2) Background selection (BS), where 95% of mutations are neutral and 5% are deleterious with selection coefficient obtained from a gamma distribution with shape parameter 0.2 and mean value *s* = –0.02 and additive gene action. (3) Selective Sweeps (SS), where 99.9% of mutations are neutral and 0.1% are assumed to be advantageous with effect obtained also from a gamma distribution with shape parameter 0.2 and mean value *s* = 0.02 and additive gene action. (4) Heterozygote advantage (overdominance) for fitness, where 99.99% of mutations are neutral and 0.01% are assumed to be advantageous with effect *s* = 0.02 and dominance coefficient *h* = 1.5. In the absence of selection and for random mating, the expected effective population size from the variance of family sizes is *N*_*eVk*_ = *N*, the number of breeding individuals. With self-fertilization with a proportion *β* = 0.5 of selfed matings, *N*_*eVk*_ is expected to be *N*_*eVk*_ = *N*/(1 + *α*), where *α* = *β* /(2 –*β*) (see, e.g., Caballero [4], p. 101), i.e., *N*_*eVk*_ = 3*N*/4.

To investigate the heterogeneity of the genome in recombination rates, the simulated sequence was divided in 70 regions of equal length and the particular rate of recombination for each region was randomly chosen from the distribution of recombination rates observed in analogous genome windows of the human genome (S6 Fig). Each simulation was run for up to 100 replicates.

### Analysis of human genomes

Data comes from the genomic sequencing of samples from two human populations: 99 individuals from a Finnish population [46], with a total of about 9.4 million SNPs, and 16 individuals from a Koryaks´ population [47], with about 4.6 million SNPs. The Koryaks population data coordinates, in genome version hg18, were converted to hg19 using liftOver UCSC tool [53]. In the process, 65,088 variants were not found and were excluded from the analyses. However, a large number of SNPs is available in both populations, allowing the study of relatively small regions of the genome. Only autosomal chromosomes were taken into account. Genomic data was divided in 2 cM regions in which local *N*_*e*_ and other genetic variables were estimated to investigate the correlations between one another. For an accurate estimation of linkage disequilibrium *N*_*e*_, only regions with more than 250,000 pairs of SNPs were considered. Telomeric regions shorter than 2 cM were also removed from the analysis. In addition, regions with extremely large *N*_*e*_ estimates (> 100,000) or negative ones were also excluded (see the distribution of *N*_*eLD*_ values for genomic regions in S4 Fig). Thus, following these criteria, 120 and 140 regions were excluded from the analyses of Finnish and Koryaks data, respectively, and the final number of regions analysed was 1,621 and 1,180, respectively.

### Estimation of *N*_*e*_

The software GONE was used to obtain historical estimates of linkage disequilibrium *N*_*e*_ (*N*_*eLD*_) using all pairs of SNPs available from simulation data at distances between *c* = 0.5 and 0.001 Morgans (M) in samples of 100 individuals. The software MSMC [18] and Relate [19] were applied to the same data, except that only samples of four randomly sampled individuals were analysed with MSMC because of computation time restrictions with this software. MSMC version 2 (downloaded in December 2019) was used with the “fixedRecombination” flag, as recommended by the user´s guide. Since the software needs several chromosomes to be run, sets of 10 replicates were run and considered as chromosomes. Relate (downloaded in December 2019) was run without monomorphic SNPs, providing the mutation rate of the simulations, the number of haplotypes of the sample, a seed, 300 bins and a threshold value of minimum mutations per tree from 50 to 30 depending on the simulation scenario. It was run for each simulation replicate and the results were averaged over replicates.

For the analysis of specific genomic regions with real data, *N*_*eLD*_ estimates were directly obtained with equations S4b and S5 of the Supplemental Material of Santiago et al. [13], which applies to the scenario of constant population size of diploid populations when the genetic phase of genotypic data is unknown. In this case, because SNPs in the regions are necessarily at relatively low genetic distances, pairs of SNPs at distances ranging between 1/50 and 1/100 M were considered in order to obtain at least 250,000 pairs per genomic region. The software Relate and MSMC were not used in these analyses, as they are assumed to apply only to historical estimates of *N*_*e*_.

### Estimation of other genomic variables

Recombination rates (*RR*; in cM/Mb) between all pairs of consecutive SNPs for each of the genomic regions were obtained from the human genetic map [34] and averaged for each region. Estimates of the background selection statistic (*B*) [54] were obtained for each site and averaged for each genomic region. A reduction in neutral diversity at a given genomic region is a function of the intensity of purifying selection and the rate of recombination, as the impact of selection on reducing diversity is higher in tight linkage regions [22,26]. The *B* statistic measures the impact of background selection on nucleotide diversity. Thus, it fluctuates between one (no background selection affecting diversity) and zero (almost complete exhaustion of diversity as a result of background selection), with an average for the human autosomal genome of about 0.74–0.81 [54].

Average nucleotide diversity (*π*), proportion of polymorphic sites (*P*) and minor allele frequency (MAF) of SNPs were calculated for each genomic region. The number of Loss of Function (LoF) and missense variants in each genomic region were also obtained using data from the 0.3.1 version of the ExAC browser [55], downloaded on 14th October 2019. Only high confidence variants were taken into account. The gene density of each genomic region was obtained using the RefSeq database [56]. When a gene spanned over different regions, we considered it to be in the region were its middle point was located. Only genes with a straightforward chromosome code were used (e.g. NC_000001.10 corresponding with chromosome 1).

## Supporting information

S1 Fig Estimates of effective population size from linkage disequilibrium (*N*_*eLD*_, sample size of *n* = 100 individuals), Relate (*N*_*eRelate*_, *n* = 100), MSMC (*N*_*eMSMC*_, *n* = 4), and from nucleotide diversity (*π*), this latter calculated as *N*_*eπ*_ = *π*/(4*μ*), where *μ* is the nucleotide mutation rate, for scenarios with different recombination rates (*RR* in cM/Mb) uniform across the genome. Simulations assume a fixed population size of *N* = 1,000 individuals with partial self-fertilization (50% of selfed progeny) under background selection and selective sweeps (see main text for mutational parameters), with constant mutation rate *μ* = 10^−8^ per base per generation. Estimates were obtained including windows of recombination rate between pairs of SNPs ranging from *c* = 0.0025 to 0.0250 for *N*_*eLD*_ and averaging historical estimates of *N*_*e*_ between generations 150 to 350 for *N*_*eRelate*_ and *N*_*eMSMC*_. Error bars represent one standard error above and below the mean of the simulation replicates. *N*_*eLD*_ estimates were obtained pooling all replicates to speed up computation.
(TIFF)

S2 Fig Estimates of effective population size from linkage disequilibrium (*N*_*eLD*_, sample size of *n* = 100 individuals), Relate (*N*_*eRelate*_, *n* = 100), MSMC (*N*_*eMSMC*_, *n* = 4), and from nucleotide diversity (*π*), this latter calculated as *N*_*eπ*_ = *π*/(4*μ*), where *μ* is the nucleotide mutation rate, for scenarios with different recombination rates (*RR* in cM/Mb) uniform across the genome. Simulations assume a fixed population size of *N* = 1,000 individuals with random mating assuming a model of heterozygote advantage (overdominance) for fitness (see main text for mutational parameters), with constant mutation rate *μ* = 10^−8^ per base per generation. Estimates were obtained including windows of recombination rate between pairs of SNPs ranging from *c* = 0.0025 to 0.0250 for *N*_*eLD*_ and averaging historical estimates of *N*_*e*_ between generations 150 to 350 for *N*_*eRelate*_ and *N*_*eMSMC*_. Error bars represent one standard error above and below the mean of the simulation replicates. *N*_*eLD*_ estimates were obtained pooling all replicates to speed up computation.
(TIFF)

S3 Fig Spearman’s correlation coefficient (*r*) of the estimated genomic variables for the Finnish (over the diagonal) and the Koryaks (below the diagonal) populations. *RR*: recombination rate; *B*: B statistic; *P*: proportion of polymorphic nucleotides; MAF: Minor Allele Frequency; *LoF*: number of Loss of Function variants; *missense*: number of missense variants; *gene dens*: gene density. P-values: * < 0.05, ** <0.01, *** <0.001.
(TIFF)

S4 Fig Distribution of estimates of linkage disequilibrium effective size (*N*_*eLD*_) for different genomic regions using data from Finnish and Koryaks populations. (TIFF)

S5 Fig Values of nucleotide diversity (*π*) for genomic regions with different average recombination rate (*RR*) using data from the Finnish and Koryaks populations. The lines indicate linear regressions.
(TIFF)

S6 Fig Distribution of mean recombination rate values for 2 cM genomic windows obtained from Myers *et al*. (2005) genetic map. Recombination rates for genomic Windows used in the simulations were randomly obtained from this distribution.
(TIFF)
